# Editorial

**Published:** 1898-11

**Authors:** 


### Editorial.

Dr. F. Bartow McRae, a former resident of Atlanta, died at Lumber City, Ga., November 17.

One Wm. Smith, an osteopath, has brought suit against the *Medical Age* of Detroit, Michigan, for $25,000 damages for alleged defamation of character. The publisher of the *Age*, Mr. Warren, promises to fight the suit *a l'outrance.* As Mr. Warren is on the side of the medical profession, he deserves the support of the medical press in his war against irregulars. We have no doubt of the outcome of the preposterous suit.

### INCOMPLETE COITION.

Apologies are due the reader for the introduction of rather a nasty subject. Ten years ago such a topic would have been tabooed, but now progress or retrogression, as the case may be, may say with the man of Destiny, *vous avons change tout cela.* It would seem from the increased frequency of allusions in the medical press to incomplete coition, or what the Germans call coitus reservatus vel interruptus, and which means withdrawal of the male member from the vagina before ejaculation, that more than usual attention is now being claimed for it.

It is said to be productive of numerous ill consequences to both participants in the act.

In the man, sooner or later, more or less profound symptoms of neurasthenia present themselves, while in the woman the pernicious effects of the practice are evidenced in a condition of general ill health and in pelvic congestion. The rationale of the influence of interrupted coition upon the woman is that erythism is aroused to a certain point without being brought to completion, so that she is not only deprived of the relaxing effect upon the congested organs concerned consequent upon that explosion of energy known as the orgasm, but of the tonic (sic) properties of the semen. One writer says: "When I go in a house and see a husband sleek, fat, jolly and hearty, and the wife pale, anæmic and nervous, if she has no definite disease, I can easily guess the cause—incomplete coition."

Now, take the facts on the other side: Is there any physiological difference on the part of the male between intra and extra vaginal ejaculation? In either instance the sexual act is complete. There is no interchange of secretion between the male and female organs concerned in the act by means of which there is a mutual replacement of that lost. There may be a mental sense of incompleteness; physically there is none.

If a man make demands upon his wife that are inordinately frequent it is convincing evidence that the natural polygamous instincts of the male of our species will lead him to find his pleasure outside of its legitimate field, and as a consequence almost inevitable will come that arch destroyer of human happiness, gonorrhœa. We know too well the far-reaching malevolent influence upon women of neglected gonorrhœa in their consort. Then why not look to that as the cause of the thin, nervous, worried, careworn looking wife than to the passing fad of the moment?

The old and well established tubal and ovarian disease of gonorrhœa origin, the lacerated cervices, endometritis, subinvolution are more often the factors that bring about premature senility in women than unorthodox methods of coition.

Incomplete coition is unnatural and abhorrent, but sentimentalists will have a difficult task in suppressing a practice which has such a fixed position in the economics of procreation.

